# Genetic variants in *COL11A2* of lumbar disk degeneration among Chinese Han population

**DOI:** 10.1002/mgg3.524

**Published:** 2018-12-08

**Authors:** Xuejun Yang, Haiyu Jia, Wenhua Xing, Feng Li, Manglai Li, Ke Sun, Yong Zhu

**Affiliations:** ^1^ Department of Spine (Thoracic and Vertebra) The Second Affiliated Hospital of Inner Mongolia University Hohhot China; ^2^ The Affiliated Hospital of Inner Mongolia Medical College Hohhot China; ^3^ Inner Mongolia Medical University Hohhot China

**Keywords:** Chinese Han population, *COL11A2*, lumbar disk degeneration, Type XI collagen

## Abstract

**Background:**

Lumbar disk disease (LDD) is a common musculoskeletal disorder. Several predisposing genetic and environmental risk factors have been established for symptomatic LDD.

**Methods:**

We conducted a case–control association study to investigate the role of the *COL11A2* gene in LDD. Genotyping of 384 Chinese Han LDD patients and 384 Chinese Han controls was made for six single‐nucleotide polymorphisms (SNPs) from *COL11A2* by Agena Massarray. We evaluated these SNPs association with LDD using the chi‐square test and genetic model analysis.

**Results:**

The strongest associations with LDD were observed for polymorphisms in rs2071025. Carriers of “A” allele had an increased risk of LDD (OR = 1.47, 95% CI = 1.20–1.80, *p* = 0.0002) as compared with the “G” allele in allele model. We found that rs2071025 were associated with LDD in female and male from the stratification analyses (*p* < 0.05). Genetic models showed that rs986522(C) significantly increased the risk of LDD in female; however, in males, we did not find significant associations between the rs986522 and LDD risk.

**Conclusion:**

This study showed a genetic association with *COL11A2* polymorphism in individuals with LDD. These data may provide novel insights into the pathogenesis of LDD, although further studies with larger numbers of participants worldwide are needed for validation of our conclusions.

## 1 INTRODUCTION

Lumbar disk degeneration (LDD) is one of the main causes of low back pain. LDD is characteristic of disk space narrowing and osteophyte growth at the circumference of the disk (Andersson, (1999)). Degeneration of the intervertebral disk is a process that begins early in life and is a consequence of various intrinsic and extrinsic factors as well as of normal aging (Phillips, (2006)). Known environmental risk factors for LDD included body mass index (BMI) and heavy physical loading (Battié & Videman, 2006), although their effect is weak in comparison with heredity (Battié, Videman, Levälahti, Gill, & Kaprio, 2008). Environmental factors seem to explain only a minor part of individual variation in pathologic changes in the disk, while the major part remains unexplained (Frymoyer, 1992; Wang & Battié, 2014). LDD has been shown to be heritable, with estimates of 65%–80% (Battié, Levalahti, Videman, Burton, & Kaprio, 2008), and so, a considerable proportion of the variance in LDD is explained by genetic factors (Ala‐Kokko, 2002). Twin studies demonstrated 74% heritability on the basis of magnetic resonance imaging (MRI) of the spine (Matsui et al., 1998). Also, genetic association studies have identified a number of risk factors. Yet to date, candidate gene studies have detected only a small number of convincing associations of genetic variants with LDD.

To date, several gene loci associated with human disk degeneration have been identified (Toktaş et al., 2015; Yi, Egan, & Wang, 2016). The first polymorphisms associated with LDD were two variations in the Vitamin D receptor gene (Videman et al., 1998). Subsequently, variations in the genes involved in inflammation, extracellular matrix components, and protein metabolism have been reported as associating with LDD (Hu, Xu, & Le, 2015; Lv et al., 2016; Willems et al., 2016). The *COL11A1*,* COL11A2*, and *COL11A3* genes encode α chains of type XI collagen (COLXI), a member of the fibrillar collagen subgroup. Type XI collagen is a cartilage‐specific ECM protein essential for cartilage collagen fibril formation and for ECM organization (Blaschke, Eikenberry, Hulmes, Galla, & Bruckner, 2000; Gregory et al., 2000). It was reported that chondrodysplasia is an autosomal recessive hereditary disease, caused by mutation of type XI collagen genes, and mutations in type XI collagen caused various types of chondrodysplasias in human, including Stickler syndrome type II. These human mutations were in vivo evidence that collagen genes are critical for cartilage formation.

However, it is not known whether the *COL11A2* gene contributes to LDD. This study was made to assess the association between relevant candidate SNPs polymorphisms and LDD risk. Focusing on Chinese Han populations can be invaluable in determining genetic predisposition to LDD.

## 2 MATERIAL AND METHODS

### 2.1 Study population

All subjects were Chinese Han who visited the participating hospitals and received medical examinations. We recruited 384 case patients with LDD and 384 control subjects. The mean ages of the case and control groups were 49.94 and 50.49 years, respectively. All LDD case patients had radiographic examination, including functional four direction images and magnetic resonance imaging (MRI) (sagittal and axial images obtained using a 1.5‐T imaging system), revealed positive findings indicating disk herniation. We excluded from the study individuals with spinal canal stenosis, spondylolisthesis, spondylitis, synovial cysts, spinal tumor, and trauma. We also excluded those who had occupational and/or habitual risk factors, such as heavy manual laborers, occupational drivers, and heavy smokers. The control group included patients with and without clinically low back pain, but with no radiographically evident degenerated disks. All procedures performed in this study were in accordance with the 1964 Helsinki declaration and its later amendments. We obtained informed consent from each subject, as approved by the Second Affiliated Hospital of Inner Mongolia Medical University and The Hohhot First Hospital.

### 2.2 SNP genotyping

We selected *COL11A2* gene six SNPs for analysis from the NCBI database. And each SNPs had minor allele frequency (MAF) of >5% in Chinese Han population. DNA was extracted from whole blood were used the GoldMag‐Mini Whole Blood Genomic DNA Purification Kit (GoldMag Co. Ltd., Xi'an City, China). Genotypes for SNPs were determined by Agena MassARRAY (Agena Bioscience). We used a NanoDrop 2000 (Gene Company Limited) to measure DNA concentrations. We used Agena MassARRAY Assay Design 3.0 Software to design a Multiplexed SNP MassEXTEND assay (Gabriel, Ziaugra, & Tabbaa, 2009). The PCR primers for each SNP are shown in Table 2. Data management and analysis were performed using the Sequenom Typer 4.0 Software (Gabriel et al., 2009; Thomas et al., 2007).

### 2.3 Statistical analysis

Chi‐square tests were used to compared cases with controls for allelic and genotypic frequencies. The odds ratio (OR) and its 95% CI were calculated. We used a permutation test to adjust significance in the analysis of association between the *COL11A2* SNPs and LDD. Linkage disequilibrium (LD) measures and haplotype blocks were estimated with Haploview (version 4.2). Haplotype frequency estimation and haplotype genetic associations were also analyzed. The level of statistical significance was set at a 0.05 for nominal association.

## 3 RESULTS

Table 1 shows sample size and demographic characteristics of 384 case patients with LDD and 384 control subjects in our study. There were not any differences in the age and gender between the LDD groups, and without LDD individuals. Allele frequencies and *COL11A2* basic information gene are shown in Table 2. For all polymorphisms of the collagen genes, the overall observed genotype frequencies were in Hardy–Weinberg equilibrium. The strongest associations with LDD were observed for polymorphisms in rs2071025. The carriers of “A” allele had an increased risk of LDD (OR = 1.47, 95% CI = 1.20–1.80, *p* = 0.0002) as compared with the “G” allele.

**Table 1:** Characteristics of the study population

	Case (*N*)	Control (*N*)	*p*
Total	384	384	
Gender			
Female	156	228	0.769
Male	160	224	
Age (years)			
Mean	49.94	50.49	0.110

Note

*p*‐value ≤0.05 indicates statistical significance.

**Table 2:** Basic information of candidate SNPs and associations with LDD risk

SNP	Gene	Chr	Alle (A/B)	MAF (case)	MAF (control)	HWE (*p*)	OR	95%CI	*p*
rs756441	*COL11A2*	6	A/G	0.445	0.436	0.756	1.04	0.85–1.27	0.714
rs17214944	*COL11A2*	6	G/A	0.081	0.052	0.613	1.60	1.06–2.41	0.204
rs3129207	*COL11A2*	6	C/G	0.529	0.496	0.838	1.14	0.93–1.39	0.202
rs9380350	*COL11A2*	6	T/C	0.413	0.439	0.918	0.90	0.73–1.10	0.298
rs986522	*COL11A2*	6	C/G	0.217	0.197	0.014	1.14	0.89–1.45	0.314
rs2071025	*COL11A2*	6	A/G	0.471	0.378	0.914	1.47	1.20–1.80	0.0002*

Note

CI: confidence interval; HWE: Hardy–Weinberg equilibrium; MAF: minor allele frequency; OR: odds ratio; SNPs: single‐nucleotide polymorphisms.

*p‐*value was calculated by Pearson's chi‐square test.

*p‐value <0.05 indicates statistical significance.

Next, we assumed that the major allele of each SNP was a reference allele and analyzed the association between each variant and LDD under four genetic models (Table 3). Three susceptibility SNPs were considered to be associated with LDD risk after the adjustment. The minor allele “G” of rs17214944 was associated with increased risk of LDD under dominant model (OR = 1.56, 95%CI = 1.01–2.39, *p* = 0.042) and log‐additive model (OR = 1.60 95%CI = 1.06–2.43, *p* = 0.024). The CC genotype of the polymorphism rs986522 was associated with increased of LDD (OR = 2.66 95%CI = 1.10–6.45, *p* = 0.022).The minor allele “A” of rs2071025 was associated with increased risk of LDD under log‐additive model (OR = 1.45, 95%CI = 1.18–1.77, *p* = 3.00E‐04). The relationship of *COL11A2* haplotypes with the risk of developing LDD was also evaluated; however, we did not find any SNPs were association with LDD risk.

**Table 3:** Genotypic model analysis of relationship between SNPs and LDD risk

SNP	Model	Genotype	Control	Case	OR (95% CI)	*p*‐value	AIC	BIC
rs17214944	Codominant	A/A	344 (89.6%)	325 (84.6%)	1	0.025*	1,066.8	1,090.1
		A/G	40 (10.4%)	56 (14.6%)	1.47 (0.96–2.28)			
		G/G	0 (0%)	3 (0.8%)	NA (0.00‐NA)			
		A/A	344 (89.6%)	325 (84.6%)	1	0.042*	1,068.1	1,086.7
		A/G‐G/G	40 (10.4%)	59 (15.4%)	1.56 (1.01–2.39)			
	Recessive	A/A‐A/G	384 (100%)	381 (99.2%)	1	0.039*	1,067.9	1,086.5
		G/G	0 (0%)	3 (0.8%)	NA (0.00‐NA)			
	Log‐additive	—	—	—	1.60 (1.06–2.43)	0.024*	1,067.1	1,085.7
rs986522	Codominant	G/G	240 (62.5%)	235 (61.2%)	1	0.072	1,069	1,092.2
		C/G	137 (35.7%)	131 (34.1%)	0.98 (0.72–1.32)			
		C/C	7 (1.8%)	18 (4.7%)	2.64 (1.08–6.44)			
	Dominant	G/G	240 (62.5%)	235 (61.2%)	1	0.71	1,072.1	1,090.6
		C/G‐C/C	144 (37.5%)	149 (38.8%)	1.06 (0.79–1.41)			
	Recessive	G/G‐C/G	377 (98.2%)	366 (95.3%)	1	0.022*	1,067	1,085.6
		C/C	7 (1.8%)	18 (4.7%)	2.66 (1.10–6.45)			
	Log‐additive	–	–	–	1.15 (0.89–1.48)	0.3	1,071.1	1,089.7
rs2071025	Codominant	G/G	148 (38.5%)	115 (29.9%)	1	7.00E‐04*	1,059.7	1,082.9
		G/A	182 (47.4%)	176 (45.8%)	1.25 (0.91–1.72)			
		A/A	54 (14.1%)	93 (24.2%)	2.21 (1.46–3.35)			
	Dominant	G/G	148 (38.5%)	115 (29.9%)	1	0.012*	1,065.9	1,084.5
		G/A‐A/A	236 (61.5%)	269 (70%)	1.47 (1.09–1.98)			
	Recessive	G/G‐G/A	330 (85.9%)	291 (75.8%)	1	4.00E‐04*	1,059.5	1,078.1
		A/A	54 (14.1%)	93 (24.2%)	1.95 (1.34–2.82)			
	Log‐additive	–	–	–	1.45 (1.18–1.77)	3.00E‐04*	1,059	1,077.6

Note

AIC: Akaike information criterion; BIC: Bayesian information criterion; CI: confidence interval; OR: odds ratio; SNPs: single‐nucleotide polymorphisms.

*p‐value <0.05 indicates statistical significance.

Finally, we used the stratification analyses, it is found in Table 4 that rs2071025 were associated with LDD in female and male. Genetic models showed that rs986522(C) significantly increased the risk of LDD in female; however, in males, we did not find significant associations between the rs986522 and LDD risk.

**Table 4:** Association between the *COL11A2* SNPs and LDD risk by stratification analysis

SNP	Gene	Alle (A/B)	MAF				HWE		OR (95%CI)		*p*	
			Male (case)	Male (control)	Female (case)	Female (control)	Male	Female	Male	Female	Male	Female
rs756441	*COL11A2*	A/G	0.46	0.47	0.42	0.39	0.59	1.00	0.98 (0.75–1.27)	1.13 (0.86–1.56)	0.856	0.444
rs17214944	*COL11A2*	G/A	0.09	0.06	0.07	0.04	1.00	1.00	1.60 (0.96–2.67)	1.58 (0.79–3.16)	0.067	0.195
rs3129207	*COL11A2*	C/G	0.45	0.47	0.50	0.45	0.69	0.26	0.93 (0.71–1.20)	1.22 (0.89–1.67)	0.563	0.209
rs9380350	*COL11A2*	T/C	0.39	0.40	0.44	0.49	0.89	0.63	0.96 (0.74–1.25)	0.82 (0.60–1.13)	0.765	0.223
rs986522	*COL11A2*	C/G	0.22	0.20	0.21	0.20	0.14	0.04	1.18 (0.86–1.62)	1.07 (0.73–1.58)	0.315	0.720
rs2071025	*COL11A2*	A/G	0.49	0.40	0.44	0.35	0.26	0.08	1.45 (1.11–1.89)	1.49 (1.08–2.06)	0.006*	0.014*

Note

CI: confidence interval; HWE: Hardy–Weinberg equilibrium; OR: odds ratio; SNP: single‐nucleotide polymorphism.

*p ≤ 0.05 indicates statistical significance.

## 4 DISCUSSION

Disk degeneration is presented as a common multifactorial and multigenic condition (Battié & Videman, 2006). At present, some of genes have been found associate with LDD (Jiang et al., 2016; Liu et al., 2016; Omair et al., 2016). This study of Chinese Han population has revealed that the association between collagen gene (*COXL11A2*) polymorphisms and LDD risk. We found the strongest associations with LDD were observed for polymorphisms in rs2071025.

Type XI collagen (COL11), a quantitatively minor component of ECM, is important for cartilage collagen fibril formation and ECM organization (Mio et al., 2007). COL11 is comprised of a1 (XI), a2 (XI), and a3 (XI) chains, which are encoded by genes *COL11A1* (1p21.1), *COL11A2* (6p21.3), and *COL2A1* (12q13.11‐q13.2), respectively (Luo & Karsdal, 2017). There are several different *COL11* single‐nucleotide polymorphisms (SNPs) that have been associated with degeneration; however, none have so far been replicated in other populations. And reported that the *COL11A2* gene is related to the development of LDD (Noponenhietala et al., 2003; Virtanen et al., 2007). Noponenhietala et al. (2003) found in rs1800587 of *COL11A2* gene individual carrying the risk T allele had an increased risk of developing degenerative lumbar spinal stenosis, which may relate to underlying degeneration. Solovieva et al. (2006) studied an in intron 9 (A/G) of *COL11A2* and found that at least one G allele was associated with an increased risk of compared to those without this polymorphism in 135 Finnish men. Videman et al. (2009) of 588 Finnish men studies found that three *COL11A2* polymorphisms (rs2072915, rs9277933, rs2076311) were associated with MRI‐defined disk bulging and signal intensity, respectively. The function of these polymorphisms is not yet clear. They may produce unstable transcripts of the disease‐associated allele. Instability would lead to decreasing functional collagen and subsequent degeneration (Mio et al., 2007). Our study selected six SNPs rs756441, rs17214944, rs3129207, rs9380350, rs986522, and rs2071025. However, these SNPs did not find any reports previous studies.

**Table 5:** Genotypic model analysis of relationship between SNPs and LDD risk by gender stratification

SNP	Model	Genotype	Male				Female			
			Control	Case	OR (95% CI)	*p*‐value	Control	Case	OR (95% CI)	*p*‐value
rs986522	Codominant	G/G	141 (63%)	134 (58.8%)	1	0.52	99 (61.9%)	101 (64.7%)	1	0.019*
		C/G	78 (34.8%)	86 (37.7%)	1.16 (0.79–1.72)		59 (36.9%)	45 (28.9%)	0.74 (0.46–1.20)	
		C/C	5 (2.2%)	8 (3.5%)	1.73 (0.55–5.48)		2 (1.2%)	10 (6.4%)	5.10 (1.07–24.18)	
	Dominant	G/G	141 (63%)	134 (58.8%)	1	0.35	99 (61.9%)	101 (64.7%)	1	0.58
		C/G‐C/C	83 (37%)	94 (41.2%)	1.20 (0.82–1.75)		61 (38.1%)	55 (35.3%)	0.88 (0.55–1.40)	
	Recessive	G/G‐C/G	219 (97.8%)	220 (96.5%)	1	0.39	158 (98.8%)	146 (93.6%)	1	0.011*
		C/C	5 (2.2%)	8 (3.5%)	1.64 (0.52–5.13)		2 (1.2%)	10 (6.4%)	5.65 (1.20–26.56)	
	Log‐additive	–	–	–	1.21 (0.86–1.70)	0.28	–	–	1.07 (0.72–1.60)	0.72
rs2071025	Codominant	G/G	85 (38%)	63 (27.6%)	1	0.028*	63 (39.4%)	52 (33.3%)	1	0.0036*
		G/A	99 (44.2%)	106 (46.5%)	1.49 (0.97–2.29)		83 (51.9%)	70 (44.9%)	1.01 (0.62–1.65)	
		A/A	40 (17.9%)	59 (25.9%)	1.98 (1.18–3.34)		14 (8.8%)	34 (21.8%)	3.05 (1.46–6.36)	
	Dominant	G/G	85 (38%)	63 (27.6%)	1	0.016*	63 (39.4%)	52 (33.3%)	1	0.27
		G/A‐A/A	139 (62%)	165 (72.4%)	1.63 (1.09–2.44)		97 (60.6%)	104 (66.7%)	1.30 (0.81–2.07)	
	Recessive	G/G‐G/A	184 (82.1%)	169 (74.1%)	1	0.052	146 (91.2%)	122 (78.2%)	1	8.00E−04*
		A/A	40 (17.9%)	59 (25.9%)	1.57 (0.99–2.48)		14 (8.8%)	34 (21.8%)	3.03 (1.54–5.97)	
	Log‐additive	–	–	–	1.42 (1.09–1.83)	0.008*	–	–	1.52 (1.09–2.12)	0.012*

Note

CI: confidence interval; OR: odds ratio; SNPs: single‐nucleotide polymorphisms.

*P‐value <0.05 indicates statistical significance.

## 5 CONCLUSIONS

Genetic polymorphisms may affect the susceptibility to an organism for the risk factors. This may explain why the population of the same risk factors only some of these develop LDD. Our results suggest that the rs2071025 G allele plays a minor role in LDD of the Chinese Han population.

## CONFLICT OF INTEREST

The authors declare no conflicts of interest.
